# Somatostatin in inflammatory bowel disease

**DOI:** 10.1080/09629359791424

**Published:** 1997-12

**Authors:** J. D. Van Bergeijk, J. H. P. Wilson

**Affiliations:** 1 Department of Gastroenterology/Internal Medicine University Hospital Dijkzigt Rotterdam The Netherlands

## Abstract

Intestinal inflammation is controlled by various immunomodulating cells, interacting by molecular mediators. Neuropeptides, released by enteric nerve cells and neuroendocrine mucosa cells, are able to affect several aspects of the general and intestinal immune system, with both pro- as well as anti-inflammatory activities. In inflammatory bowel disease (IBD) there is both morphological as well as experimental evidence for involvement of neuropeptides in the pathogenesis. Somatostatin is the main inhibitory peptide in inflammatory processes, and its possible role in IBD is discussed.


Mediators of Inflammation, **6**, 303–309 (1997)

### Somatostatin in inflammatory bowel disease

J. D. van Bergeijk[CA] and J. H. P. Wilson

Department of Gastroenterology/Internal Medicine, University Hospital Dijkzigt, Rotterdam, The Netherlands

[CA]Corresponding Author
Tel: (+31) 10 4635253
Fax: (+31) 10 4634682

**Intestinal inflammation is controlled by various immunomodulating cells, interacting by molecular mediators. Neuropeptides, released by enteric nerve cells and neuroendocrine mucosa cells, are able to affect several aspects of the general and intestinal immune system, with both pro- as well as anti-inflammatory activities. In inflammatory bowel disease (IBD) there is both morphological as well as experimental evidence for involvement of neuropeptides in the pathogenesis. Somatostatin is the main inhibitory peptide in inflammatory processes, and its possible role in IBD is discussed.**

**Key words:** Inflammatory bowel disease, Neuropeptide, Somatostatin, Gastrointestinal immune system

### Introduction

Ulcerative colitis and Crohn's disease are both characterized by chronic, relapsing intestinal inflammation. The aetiology of both forms of inflammatory bowel disease (IBD) is still unknown, despite intensive research in two main areas—abnormal regulation of the local immune response and exogenous factors, including infectious agents. The gastrointestinal immune system protects the organism against toxins, microorganisms and dietary antigens within the gut lumen. It is generally assumed that luminal stimuli presented to intestinal mucosa activate intra-epithelial immunocytes resulting in release of mediators of inflammation. In IBD an inflammatory cascade is initiated and, due to insufficient or inappropriate immune reactions, intestinal damage results.[1,2] The reasons for inappropriate immune activation are unknown. Central roles are currently ascribed in this process to activated intestinal macrophages and T-lymphocytes[2] in combination with an imbalance between pro- and contra-inflammatory T-cells.[3–5] Treatment of IBD is aimed at reducing the production or action of mediators of inflammation.

Delicate interactions of intestinal mucosal epithelium, smooth muscle cells, gut wall blood vessel endothelium, immunocytes and enteric nerve cells contribute to and regulate intestinal inflammatory changes. These processes are mediated by various chemical messengers, most of which are produced by lymphocytes, granulocytes and macrophages. Recent studies on aetiopathogenesis and treatment in IBD have concentrated on these immunocyte products which include cytokines, eicosanoids and adhesion molecules. The contribution of other elements of the gastrointestinal immunological defence system to chronic intestinal inflammation is less well known. A potentially interesting area is the immunoregulatory role of enteric nerves and neuroendocrine cells. Neuropeptides, like substance P (SP), somatostatin (SMS), vasoactive intestinal peptide (VIP) and calcitonin gene related peptide (CGRP), are the molecular mediators of neuroregulation of the intestinal immune system, providing for interactions between nervous system and immunocytes. SMS is a key inhibiting factor of many biological processes. In this review the role of SMS as neuroimmune modulator in IBD will be highlighted, together with its possible future use in the treatment of IBD.

### Neuroinflammation, Neuropeptides and Intestinal Inflammation

The concept of neuroimmune interaction is in part derived from older clinical observation that inflammatory processes are influenced by emotional or physical stress. The immune system is subject to central nervous control and Pavlovian responses.[6] Although IBD patients do not have more emotional difficulties or psychosocial stress compared with a normal population, disease activity and response to therapy are certainly influenced by the state of mental well-

© 1997 Rapid Science Publishers being.[7] The interaction between nervous system and the intestinal immune system is probably mediated by neuropeptides derived from enteric nerves and neuroendocrine cells.[8–11]

Membrane-bound neuropeptide receptors are found on several immunocytes, including T-lymphocytes and monocytes.[6,9,12–16] Various neuropeptides affect intestinal lymphocyte function and several cells of the intestinal immune system also produce neuropeptides, suggesting a local immunoregulatory task.[17–20] Migration of immunocytes into the intestinal mucosa is affected by neuropeptides.[21]

In addition there are several morphological arguments that suggest neuropeptide involvement in intestinal mucosal immunity. Intestinal mucosa contains SP, VIP and SMS immunoreactive nerve fibres and co-localization is common.[22,23] Neuropeptides show a specific distribution along the gut. Transmural distribution can be different for different neuropeptides.[24–27]

The reported studies of neuropeptide immunomodulation in the intestine need to be interpreted with some caution. Receptor binding studies often show unexpected concentration relationships and depend strongly on local conditions.[28–34] Autoradiography is a semiquantitative approach and sampling error in taking mucosal biopsies or loss of neuropeptide containing cells by inflammatory damage, may account for some of the confusing results. Structural and species specific neuropeptide receptor heterogeneity may further complicate comparison of results.[35–38] Neuropeptides are known to exert different effects at different sites. For instance skin mast cells are susceptible to SMS, whereas SMS does not induce histamine release from intestinal mast cells.[39]

Neuropeptides may be pro- or anti-inflammatory. Generally, substance P (SP) has a pro-inflammatory action. Macrophage interleukin-1 (IL-1) production and cytotoxicity is stimulated by SP[20,40] as is IL-1, TNFα and IL-6 release by human monocytes.[31] SP stimulates proliferation of and immunoglobulin synthesis by lymphocytes from spleen, mesenteric lymph nodes and Peyer's patches.[28] Moreover, in experimental infection with *Schistosoma mansoni* SP induces IFNγ production by granuloma macrophages.[41] However, a dose-dependent inhibition of immunoglobulin production of murine intestinal granuloma derived B-cells has been described.[42] Some of these pro-inflammatory effects of SP are opposed by SMS. SMS inhibits macrophage SP-induced IFNγ release.[41] SP-induced chemotaxis of neutrophils can be completely reversed by the SMS analogue octreotide.[43]

In mucosal lymphocytes from resected human colon segments, DNA synthesis as measured by $^3$H-thymidine uptake, is inhibited by low concentrations of SMS, VIP, SP and bombesin.[44] This inhibition might be principally achieved by T-cell suppression, as it is observed in lymphocytes that are stimulated by concanavalin A. The maximum inhibitory effects are obtained after 4 days of neuropeptide incubation.[44] VIP and SMS inhibit lymphocytic proliferation in a dose-dependent fashion.[17,28,29,45] The inhibitory effects of VIP and SMS on these lymphocytes are mediated by specific receptors, not by cytotoxicity.[46–48]

VIP has both pro- and anti-inflammatory effects on intestinal T-cells and macrophages, inducing IL-5 release and inhibiting macrophage adherence.[49,50] T-cell cAMP increases on stimulation by VIP, but proliferation is inhibited.[38,51] However, reactivity of granuloma B-cells and macrophages is not affected.[38,42]

Conflicting results emerge from studies in IBD patients. SP content of inflamed colon is increased, but there is a substantial overlap with normal SP content.[52,53] SP receptor upgrading is observed in inflamed areas of IBD colon.[54–57] SP receptor expression in ulcerative colitis enteric nerves has been found to be normal[57] or increased.[54] For VIP the observations are even more confusing. Whereas VIP concentration in plasma of patients with active IBD is increased,[58] colon VIP content is reported to be either decreased[53,59,60] or increased.[55,61] There is no clear relation between VIP content and disease activity.

### Somatostatin

SMS was first extracted from ovine hypothalamus as an inhibitor of growth hormone secretion.[62] SMS is a peptide hormone. There are two biologically active forms, consisting of 14 or 28 amino acids respectively. Most of the total body SMS content is stored in the digestive tract. About 75% is localized in gut and pancreas.[63,64] In stomach and pancreas SMS-14 prevails, and in the gut SMS-28.[65] SMS containing cells are found in enteric mucosa, submucosa and neural tissue.[8] More than 90% of gut SMS content is localized in the endocrine cells of the mucosa, less than 10% in enteric nerves of the muscular layer.[66,67]

Five types of SMS receptors have been discovered, each with specific binding characteristics for SMS subtypes and different SMS analogues.[68] SMS receptors are found in upper and lower parts of the normal digestive tract.[66] SMS is an inhibitor of several key functions in the body. SMS inhibits acid secretion, intestinal fluid absorption, intestinal and pancreas secretion. SMS has effects on splanchnic blood flow and gastric and intestinal motility.[66] SMS exerts its inhibitory effects by diminishing intracellular cAMP through G-protein activation. In addition, SMS impedes cellular influx of calcium. This impediment results from a direct effect on calcium channels and from increase of potassium conductance with subsequent cellular hyperpolarization.[64] Circulating native SMS has a short half-life time. Long-acting analogues like the decapeptide octreotide have been developed[69] and SMS analogues have been used to treat intractable diarrhoea, bleeding from oesophageal varices in portal hypertension, dumping syndrome and gastrointestinal fistulae.[70–74] Radioactive labelled SMS analogues serve as diagnostic tools in visualization of gastrointestinal neuroendocrine tumours.[75]

### Immunomodulatory Effects of Somatostatin

Studies on the immunomodulatory effects of SMS show several effects on T- and B-lymphocytes and macrophages. SMS receptors exist in spleen, liver, thymus and gastrointestinal lymphoid tissue, as well as on various immunocytes.[12,15,76] Immunomodulating actions of SMS were discovered as a result of its antagonizing effects of proliferation of rat lymphocytes, stimulated by hypothalamic extracts.[77] SMS inhibits responsiveness, immunoglobulin synthesis and proliferation of lymphocytes and granulocytes.[15,45,46,78–80] It reduces TNF release and cellular toxicity of stimulated rat peritoneal macrophages.[30,81] Inhibitory effects depend on local SMS concentration. Several *in vitro* studies show inhibition of proliferation of lymphoid cells at low SMS concentrations and stimulation at high levels.[46,77] In an experimental model of intestinal inflammation with *Schistosoma mansoni*, SMS as well as its analogue octreotide decrease T-cell interferon production significantly.[82]

Some studies report immunostimulating effects by SMS. T-cell proliferation is seen, also at low SMS concentrations.[83,84] In rat peritoneal macrophages SMS stimulates cytotoxic reactivity, when given in low concentrations.[81] T-cell activation in a hybridoma T-lymphocyte cell line, measured by IL-2 release, is stimulated by SMS in a dose-dependent way.[17] However, IL-2 receptor expression is inhibited by SMS in human intestinal lymphocytes.[47,48]

SMS controls inflammatory processes in *in vivo* experimental models. A reduction of inflammatory infiltrate and a diminished TNFα production occurs when SMS analogues are applied to animals in which carrageen-induced skin inflammation is established.[85] In this experiment intense SMS immunostaining was seen on leukocytes at peri-inflammatory sites. SMS reduces inflammation in experimental arthritis and ileal obstruction.[86,87] Similar beneficial effects on intestinal and colonic inflammation emerge from clinical observations in Crohn's disease and gold-induced enteritis.[88,89] SMS is able to reduce SP mediated inflammation induced by intestinal infection with *Trichinella spiralis*[90] and SP enhanced neutrophil chemotaxis.[43]

### Somatostatin in IBD

No systematic *in vivo* or *in vitro* studies on effects of SMS in IBD are available at present. Support for SMS induced immunomodulation in IBD is indirect and derived from morphological and biochemical analyses of intestinal and blood specimens from IBD patients. From several studies a correlation between SMS activity and presence and intestinal inflammation emerges.

When measured in serum total body SMS release shows a circadian rhythm.[91,92] In active ulcerative colitis a higher 24-hour amplitude, higher average serum levels and a longer meal-stimulated peak level are observed.[91,93] As the serum concentration is only a faint mirror of the mucosal events, the impact of increased secretion of SMS is obscure. Same response patterns are seen in patients with duodenal ulcer or irritable bowel syndrome.[93]

SMS containing cells and submucosal ganglion cells in surgical specimen from IBD patients can be visualized by immunohistochemical staining and quantified by counting the SMS containing cells per cm. Mucosal SMS content can be assessed by radioimmunoassay of homogenized biopsy specimen. In normal colon mucosa, SMS containing endocrine cells show the highest density in the distal parts. In active IBD this distinct difference disappears. Studies prior to the discovery of SMS showed a decrease of neuroendocrine enterochromaffin cells in diseased rectum of ulcerative colitis patients.[94,95] In ulcerative colitis there is an actual decrease in SMS containing cells, especially in the distal part of the colon.[96–99] Although these changes may be secondary to inflammatory damage to mucosal SMS containing cells, this is refuted by the fact that other mucosal neuropeptides like SP show increased levels in these cases.[98] SMS containing submucosal ganglion cells are evenly distributed along the colon in normals and IBD patients, but the number of these cells is decreased in IBD.[97] In colon epithelial cell cultures from patients with active ulcerative colitis, decreased SMS generation is observed. This loss of SMS production correlates with disease activity.[100]

In Crohn's disease the loss of SMS containing colonic cells is less evident. A tendency towards decrease of SMS positive cells with increasing disease activity has been reported.[97] No difference of SMS content is seen in mucosa or normal ileum and terminal ileitis.[98] Mucosal biopsies from inflamed jejunum in Crohn's disease show normal levels of SMS, but soluable SMS is increased.[101] This may reflect instability of SMS storage granules due to inflammation.

Several arguments for SMS involvement in mucosal inflammation emerge from scintigraphic studies. An increased density of SMS receptors is found in areas of granulomatous inflammation like tuberculosis, sarcoidosis and Wegener's granulomatosus.[102–104] From SMS receptor measurements in granulomas of murine intestinal *Schistosoma mansoni* infestation emerge the same results.[82]

High-affinity SMS receptors are found in normal jejunum, ileum and colon.[105] Apart from presence in colon mucosa and nerve plexus, SMS receptors are found in the germinal centres of colonic lymph follicles. SMS receptors are seen in gut-associated lymphatic tissue, like palatine tonsils, Peyer's patches, vermicular appendix and isolated lymphatic follicles in the colon[106] and SMS is isolated from nervous tissue in Peyer's patches.[107] The precise function of SMS in this gut-associated lymphoid tissue has not yet been settled. High receptor density is seen in the luminal parts of secondary lymph follicles, but they are absent from the corona of B-lymphoid cells. Lymphoid aggregates without a germinal centre do not show receptor activity.[106] Interaction of SMS and lymphocytes from these germinal centres is reasonable. As these receptors have high affinity for SMS, a specific immunomodulatory role of SMS is anticipated. Other indications for a direct influence of SMS on intestinal immunocytes can be derived from electron microscopic studies of the gut wall. SMS containing enteric nerve fibres are present in a very high density near intestinal lymph follicles, coming close to follicular lymphocytes.[107]

Inhibitory effects of SMS on vascular cell proliferation have been described.[108] Expression of high affinity SMS receptors is seen in intramural veins of inflamed intestinal mucosa in IBD or peri-inflammatory veins in rheumatoid arthritis.[109,110] No difference is seen in SMS receptor content of normal and inflamed tissue in mucosa, nerve plexus or lymphatic follicles. Precise cellular localization of the SMS receptors is not possible from autoradiography, due to insufficient resolution of this visual technique. Histologically these receptor positive veins are normal, but the surrounding tissue often is infiltrated by leukocytes and receptor positivity is correlated with IBD activity.[109] However, expression of SMS receptors in vessel walls could be a nonspecific response to inflammation as this phenomenon is also seen in peritumoral tissues in resected SMS receptor negative colon adenocarcinoma and other malignancies.[111,112]

In animal experimental models of inflammatory bowel disease there are several suggestions of a role of SMS in the inflammatory mucosal responses. In murine experimental colitis SMS prevents mucosal damage effectively, especially when given before the introduction of the toxin.[113] Parallel to decrease of mucosal lesions a decrease of inflammatory mediators such as leukotriene B4 and platelet activating factor is seen.

Interleukin-2 receptor expression and DNA synthesis in intestinal lamina propria lymphocytes (LPL) is reduced by SMS.[44,47,48] Proliferation of Peyer's patch derived lymphocytes is inhibited by SMS. Inhibitory effects of SMS on lymphocytic proliferation are more pronounced in intestinal derived T-cells than in splenic lymphocytes.[114] Affinity of lamina propria lymphocytes for SMS-binding was found to be 1000 times higher than peripheral blood lymphocytes in one study.[48]

However, conflicting results emerge from a study in which effects on human peripheral blood lymphocytes and intestinal LPL were compared. The effects of SMS on intestinal LPL were minimal. Although both lymphoid cells expressed high affinity SMS receptors, intestinal lymphoid proliferation was poorly inhibited by SMS. Immunoglobulin synthesis was affected in a dose-related way in both peripheral and intestinal lymphocytes.[48] The fact that these results run counter to earlier reported data, may be due to species differences and the lack of SMS receptor subtyping.

Intestinal granulomas induced by *Schistosoma mansoni* are smaller in the presence of SMS. Their output of interferon γ and immunoglobulin is diminished by SMS. As these same granulomas are also capable of producing SMS, this suggests a local immunoregulatory role for SMS.[19,82,115] This is supported by the interesting observation that intra-epithelial lymphoid cells can stimulate isolated intestinal epithelium to produce SMS.[116]

### Conclusion

From morphological studies it is clear that neuropeptides are probably involved in the control of the intestinal immune system. Immune stimulatory and inhibitory effects emerge from various *in vitro* studies and from sparse *in vivo* experiments. SMS has been shown to inhibit immunological processes at various sites following different stimuli. As SMS receptors show a high density in the gastrointestinal associated lymphoid tissue and in inflammatory granulomas in murine *Schistosoma mansoni* infestation, it seems likely that SMS and its receptors play a role in immunological events of the digestive tract. Mucosal SMS content is reduced in active IBD. The beneficial effects of SMS and SMS analogues on experimental inflammation of skin, joints and intestine and the few reports on open studies in patients with IBD suggest that SMS and SMS analogues could possibly be beneficial in IBD and should stimulate further clinical studies.

### References

1. Elson CO, McCabe RP. The immunology of inflammatory bowel disease. In: Kirsner JB, Shorter RG, eds. *Inflammatory Bowel Disease.* Baltimore: Williams & Wilkins, 1995; 203–251.
2. MacDermott RP. Alterations in the mucosal immune system in ulcerative colitis and Crohn's disease. *Med Clin North Am* 1994; **78**: 1207–1231.
3. Sartor RB. Cytokines in intestinal inflammation: pathophysiological and clinical considerations. *Gastroenterology* 1994; **106**: 533–539.
4. Niessner M, Volk BA. Altered Th1/Th2 cytokine profiles in the intestinal mucosa of patients with inflammatory bowel disease as assessed by quantitative reversed transcribed polymerase chain reaction (PT-PCR). *Clin Exp Immunol* 1995; **101**: 428–435.
5. Mayer L, Eisenhardt D. Lack of induction of suppressor T cells by intestinal epithelial cells from patients with inflammatory bowel disease. *J Clin Invest* 1990; **86**: 1255–1260.
6. Shanahan F, Anton P. Neuroendocrine modulation of the immune system. Possible implications for inflammatory bowel disease. *Dig Dis Sci* 1988; **33**: 41S–49S.
7. Calkins BM, Mendeloff AI. The epidemiology of idiopathic inflammatory bowel disease. In: Kirsner JB, Shorter RG, eds. *Inflammatory Bowel Disease.* Baltimore: Williams & Wilkins, 1995; 31–68.
8. Eliakim R, Rachmilewitz D. Inflammatory mediators and the pathogenesis of inflammatory bowel disease. *Ital J Gastroenterol* 1992; **24**: 361–368.
9. Spirinek LP, O'Dorisio MS. Modulation of immune function by intestinal neuropeptides. *Acta Oncologica* 1991; **30**: 509–517.
10. O'Dorisio MS. Neuropeptides and gastrointestinal immunity. *Am J Med* 1986; **81** (suppl 6B): 74–80.
11. Reichlin S. Neuroendocrine-immune interactions. *N Engl J Med* 1993; **329**: 1246–1253.
12. Bhathena SJ, Louie J, Schechter GP, Redman RS, Wahl L, Recant L. Identification of human mononuclear leukocytes bearing receptors for somatostatin and glucagon. *Diabetes* 1981; **30**: 127–131.
13. Beed EA, O'Dorisio MS, O'Dorisio TM, Gaginella T. Demonstration of a functional receptor for vasoactive intestinal polypeptide on Molt 4b T lymphoblasts. *Regul Pept* 1983; **6**: 1–12.
14. Elliott DE, Metwali A, Blum AM, Sandor M, Lynch R, Weinstock JV. T lymphocytes isolated from the hepatic granulomas of Schistosoma-infected mice express somatostatin receptor subtype II (SSTR2) messenger RNA. *J Immunol* 1994; **153**: 1180–1186.
15. Scicchitano R, Dazin P, Bienenstock J, Payan DG, Stanisz AM. The murine IgA secreting plasmacytoma MOPC-315 expresses somatostatin receptors. *J Immunol* 1988; **141**: 937–941.
16. Sreedharan S, Kodama KT, Peterson KE, Goetzl EJ. Distinct subsets of somatostatin receptors on cultured human lymphocytes. *J Biol Chem* 1989; **264**: 949–942.
17. Nio DA, Moylan RN, Roche JK. Modulation of T lymphocyte function by neuropeptides. Evidence for their role as local immunoregulatory elements. *J Immunol* 1993; **150**: 5281–5288.
18. Weinstock JV, Blum AM. Release of substance P by granuloma eosinophils in response to secretagogues in murine *Schistosomiasis mansoni*. *Cell Immunol* 1990; **125**: 380–385.
19. Weinstock JV, Blum AM, Malloy T. Macrophages within the granulomas of murine *Schistosoma mansoni* are a source of a somatostatin 1-14-like molecule. *Cell Immunol* 1990; **131**: 381–390.
20. Pascual DW, Bost KL. Substance P production by P388D1 macrophages: a possible autocrine function for this neuropeptide. *Immunology* 1990; **71**: 52–56.
21. Ottaway CA. Neuropeptides, neurons and mucosal lymphoid cell migration. *Monogr Allergy* 1988; **24**: 157–166.
22. Accili EA, Dhatt N, Buchan AM. Neural somatostatin, vasoactive intestinal polypeptide and substance P in canine and human jejunum. *Neurosci Lett* 1995; **185**: 37–40.
23. Pataky DM, Curtis SB, Buchan AM. The co-localization of neuropeptides in the submucosa of the small intestine of normal Wistar and non-diabetic BB rats. *Neuroscience* 1990; **36**: 247–254.
24. Crowe R, Kamm MA, Burnstock G, Lennard-Jones JE. Peptide-containing neurons in different regions of the submucous plexus of human sigmoid colon. *Gastroenterology* 1992; **102**: 461–467.
25. Ferri GL, Adrian TE, Allen JM, *et al.* Intramural distribution of regulatory peptides in the sigmoid-recto-anal region of the human gut. *Gut* 1988; **29**: 762–768.
26. Hörsch D, Fink T, Göke B, Arnold R, Büchler M, Weihe E. Distribution and chemical phenotypes of neuroendocrine cells in the human anal canal. *Regul Pept* 1994; **54**: 527–542.
27. Probert L, De Mey J, Polak JM. Ultrastructural localization of four different neuropeptides within separate populations of p-type nerves in the guinea pig colon. *Gastroenterology* 1983; **85**: 1094–1104.
28. Stanisz AM, Befus D, Bienenstock J. Differential effects of vasoactive intestinal peptide, substance P, and somatostatin on immunoglobulin synthesis and proliferations by lymphocytes from Peyer's patches, mesenteric lymph nodes, and spleen. *J Immunol* 1986; **136**: 152–156.
29. Tang SC, Braunsteiner H, Wiedermann CJ. Regulation of human T lymphoblast growth by sensory neuropeptides: augmentation of cholecystokin-induced inhibition of Molt-4 proliferation by somatostatin and vasoactive intestinal peptide *in vitro*. *Immun Lett* 1992; **34**: 237–242.
30. Chao T-C, Cheng H-P, Walter RJ. Somatostatin and macrophage function: modulation of hydrogen peroxide, nitric oxide and tumor necrosis factor release. *Regul Pept* 1995; **58**: 1–10.
31. Lotz M, Vaughan JH, Carson DA. Effects of neuropeptides on production of inflammatory cytokines by human monocytes. *Science* 1988; **241**: 1218–1221.
32. Uribe A, Alam M, Johansson O, Midtvedt T, Theodorsson E. Microflora modulates endocrine cells in the gastrointestinal mucosa of rats. *Gastroenterology* 1994; **107**: 1259–1269.
33. Miller GV, Preston SR, Woodhouse LF, Farmery SM, Promrose JN. Somatostatin binding in human gastrointestinal tissues: effect of cations and somatostatin analogues. *Gut* 1993; **34**: 1351–1356.
34. Roberts AI, Taunk J, Ebert EC. Human lymphocytes lack substance P receptors. *Cell Immunol* 1992; **141**: 457–465.
35. Patel YC, Murthy KK, Escher EE, Banville D, Spiess J, Srikant CB. Mechanism of action of somatostatin: an overview of receptor function and studies of the molecular characterization and purification of somatostatin receptor proteins. *Metabolism* 1990; **9** (suppl 2): 63–69.
36. Bell GI, Yasuda K, Kong H, Law SF, Raynor K, Reisine T. Molecular biology of somatostatin receptors. *Ciba Found Symp* 1995; **190**: 65–88.
37. Coy DH, Taylor JE. Receptor-specific somatostatin analogs: correlations with biological activity. *Metabolism* 1996; **45** (suppl 1): 21–23.
38. Weinstock JV, Blum AM, Khetarpal S. Granulomas in murine *Schistosomiasis mansoni* contain vasoactive intestinal peptide-responsive lymphocytes. *Cell Immunol* 1991; **134**: 458–472.
39. Church MK, Benyon RC, Lowman MA, Hutson PA, Holgate ST. Allergy or inflammation? From neuropeptide stimulation of human skin mast cells to studies on the mechanism of the late asthmatic response. *Agents Actions* 1989; **26**: 22–30.
40. Peck R. Neuropeptides modulating macrophage function. *Ann NY Acad Sci* 1987; **496**: 264–270.
41. Blum AM, Metwali A, Cook G, Mathew RC, Elliott D, Weinstock JV. Substance P modulates antigen-induced, IFN-gamma production in murine *Schistosomiasis mansoni*. *J Immunol* 1993; **151**: 225–233.
42. Neil GA, Blum A, Weinstock JV. Substance P but not vasoactive intestinal peptide modulates immunoglobulin secretion in murine schistosomiasis. *Cell Immunol* 1991; **135**: 394–401.
43. Kolasinski SL, Haines KA, Siegel EL, Cronstein BN, Abramson SB. Neuropeptides and inflammation. A somatostatin analog as a selective antagonist of neutrophil activation by substance P. *Arthritis Rheum* 1992; **35**: 369–375.
44. Elitsur Y, Luk GD. Gastrointestinal neuropeptides suppress human colonic lamina propria lymphocyte DNA synthesis. *Peptides* 1990; **11**: 879–884.

45. Malec P, Zeman K, Markiewicz K, Tchorzewski H, Nowak Z, Baj Z. Short-term somatostatin infusion affects T lymphocyte responsiveness in humans. *Immunopharmacology* 1989; **17**: 45–49.
46. Payan DG, Hess CA, Goetzl EJ. Inhibition by somatostatin of the proliferation of T-lymphocytes and Molt-4 lymphoblasts. *Cell Immunol* 1984; **84**: 433–438.
47. Pallone F, Fais S, Annibale B, Boirivant M, Morace S, Delle Fave G. Modulatory effects of somatostatin and vasoactive intestinal peptide on human intestinal lymphocytes. *Ann NY Acad Sci* 1990; **594**: 408–410.
48. Fais S, Annibale B, Boirivant M, Santoro A, Pallone F, Delle Fave G. Effects of somatostatin on human intestinal lamina propria lymphocytes. Modulation of lymphocyte activation. *J Neuroimmunol* 1991; **31**: 211–219.
49. Mathew RC, Cook GA, Blum AM, Metwali A, Felman R, Weinstock JV. Vasoactive intestinal peptide stimulates T lymphocytes to release IL-5 in murine *Schistosomiasis mansoni* infection. *J Immunol* 1992; **148**: 3572–3577.
50. Segura JJ, Guerrero JM, Lopez-Gonzalez MA, Calvo JR. Vasoactive intestinal peptide (VIP) inhibits substrate adherence capacity of rat peritoneal macrophages by a mechanism that involves cAMP. *Cell Adhes Commun* 1993; **1**: 213–221.
51. Metwali A, Blum A, Mathew R, Sandor M, Lynch RG, Weinstock JV. Modulation of T lymphocyte proliferation in mice infected with *Schistosoma mansoni*: VIP suppresses mitogen- and antigen-induced T cell proliferation possibly by inhibiting IL-2 production. *Cell Immunol* 1993; **149**: 11–23.
52. Goldin E, Karmeli F, Selinger Z, Rachmilewitz D. Colonic substance P levels are increased in ulcerative colitis and decreased in chronic severe constipation. *Dig Dis Sci* 1989; **34**: 754–757.
53. Koch TR, Carney JA, Go VLW. Distribution and quantitation of gut neuropeptides in normal intestine and inflammatory bowel disease. *Dig Dis Sci* 1987; **32**: 369–376.
54. Keranen U, Kiviluoto T, Jarvinen H, Back N, Kivilaakso E, Soinila S. Changes in substance P-immunoreactive innervation of human colon associated with ulcerative colitis. *Dig Dis Sci* 1995; **40**: 2250–2258.
55. Keranen U, Jarvinen H, Kiviluoto T, Kivilaakso E, Soinila S. Substance P- and vasoactive intestinal polypeptide-immunoreactive innervation in normal and inflamed pouches after restorative proctocolectomy for ulcerative colitis. *Dig Dis Sci* 1996; **41**: 1658–1664.
56. Mantyh PW, Catton M, Maggio JE, Vigna SR. Alterations in receptors for sensory neuropeptides in human inflammatory bowel disease. *Adv Exp Med Biol* 1991; **298**: 253–283.
57. Mantyh CR, Vigna SR, Bollinger RR, Mantyh PW, Maggio JE, Pappas TN. Differential expression of substance P receptors in patients with Crohn's disease and ulcerative colitis. *Gastroenterology* 1995; **109**: 850–860.
58. Duffy LC, Zielezny MA, Riepenhoff-Talty M, *et al.* Vasoactive intestinal peptide as a laboratory supplement to clinical activity index in inflammatory bowel disease. *Dig Dis Sci* 1989; **34**: 1528–1535.
59. Schulte-Bockholt A, Fink JG, Meier DA, *et al.* Expression of mRNA for vasoactive intestinal peptide in normal human colon and during inflammation. *Mol Cell Biochem* 1995; **142**: 1–7.
60. Kimura M, Masuda T, Hiwatashi N, Toyota T, Nagura H. Changes in neuropeptide-containing nerves in human colonic mucosa with inflammatory bowel disease. *Pathol Int* 1994; **44**: 624–634.
61. Bishop AE, Polak JM, Bryant MG, Bloom SR, Hamilton S. Abnormalities of vasoactive intestinal polypeptide-containing nerves in Crohn's disease. *Gastroenterology* 1980; **79**: 853–860.
62. Brazeau P, Vale W, Burgus R, *et al.* Hypothalamic polypeptide that inhibits the secretion of immunoreactive pituitary growth hormone. *Science* 1973; **179**: 77–79.
63. Patel YC, Wheatley T, Ning C. Multiple forms of immunoreactive somatostatin: comparison of distribution in neural and nonneural tissues and portal plasma of the rat. *Endocrinology* 1981; **109**: 1943–1949.
64. Shulkes A. Somatostatin: physiology and clinical applications. *Baillière's Clin Endocr Metab* 1994; **8**: 215–236.
65. Rabbani SH, Patel YC. Peptides derived by processing of rat prosomatostatin near the amino-terminus: characterization, tissue distribution, and release. *Endocrinology* 1990; **126**: 2054–2061.
66. Schusdziarra V. Somatostatin—physiology and pathophysiological aspects. In: Polak JM, Bloom SR, Wright NA, Butler AG, eds. *Basic Science in Gastroenterology. Physiology of the Gut.* Ware: Glaxo Group Research, 1984; 69–84.
67. Penman E, Wass JAH, Butler MG, *et al.* Distribution and characterization of immunoreactive somatostatin in human gastrointestinal tract. *Regul Pept* 1983; **7**: 53–65.
68. Bruns C, Raulf F, Hoyer D, Schloos J, Lubbert H, Weckbecker G. Binding properties of somatostatin receptor subtypes. *Metabolism* 1996; **45** (suppl 1): 17–20.
69. Bauer W, Briner U, Doepfner W, *et al.* SMS 201-995: a very potent and selective octapeptide analogue of somatostatin with prolonged action. *Life Sciences* 1982; **31**: 1133–1140.
70. Lamberts SWJ, van der Lely A-J, de Herder WW, Hofland LJ. Octreotide. *N Engl J Med* 1996; **334**: 246–254.
71. Battershil PE, Clissold SP. Octreotide. A review of its pharmacodynamic and pharmacokinetic properties, and therapeutic potential in conditions associated with excessive peptide secretion. *Drugs* 1989; **38**: 658–702.
72. Burroughs AK. Octreotide in variceal bleeding. *Gut* 1994; **35** (suppl 3): S23–S27.
73. Harris AG. Octreotide in the treatment of disorders of the gastrointestinal tract. *Drug Investigation* 1992; **4** (suppl 3): 1–54.
74. Owyang C. Octreotide in gastrointestinal motility disorders. *Gut* 1994; **35** (suppl 3): S11–S14.
75. Krenning EP, Kooij PPM, Pauwels S, *et al.* Somatostatin receptor: scintigraphy and radionuclide therapy. *Digestion* 1996; **57** (suppl 1): 57–61.
76. van Hagen PM, Krenning EP, Kwekkeboom DJ, *et al.* Somatostatin and the immune and haematopoetic system; a review. *Eur J Clin Invest* 1994; **24**: 91–99.
77. Pawlikowski M, Stepien H, Kunert-Radek J, Schally AV. Effect of somatostatin on the proliferation of mouse spleen lymphocytes *in vitro*. *Biochem Biophys Res Commun* 1985; **129**: 52–55.
78. Goetzl EJ, Payan DG. Inhibition by somatostatin of the release of mediators from human basophils and rat leukemic basophils. *J Immunol* 1984; **133**: 3255–3259.
79. Pawlikowski M, Stepien H, Kunert-Radek J, Zelazowski P, Schally AV. Immunomodulatory action of somatostatin. *Ann NY Acad Sci* 1987; **496**: 233–239.
80. Eglezos A, Andrews PV, Helme RD. *In vivo* inhibition of the rat primary antibody response to antigenic stimulation by somatostatin. *Immunol Cell Biol* 1993; **71**: 125–129.
81. Fóris G, Gyimesi E, Komáromi I. The mechanism of antibody-dependent cellular cytotoxicity stimulation by somatostatin in rat peritoneal macrophages. *Cell Immunol* 1985; **90**: 217–225.
82. Blum AR, Metwali A, Mathew RC, Cook G, Eliott D, Weinstock JV. Granuloma T lymphocytes in murine *Schistosomiasis mansoni* have somatostatin receptors and respond to somatostatin with decreased IFN-gamma secretion. *J Immunol* 1992; **149**: 3621–3626.
83. Nordlind K, Mutt V. Influence of beta-endorphin, somatostatin, substance P, and vasoactive intestinal peptide on the proliferative response of human peripheral blood T lymphocytes to mercuric chloride. *Int Arch Allergy Appl Immunol* 1986; **80**: 326–328.
84. Johansson O, Sandberg G. Effect of neuropeptides beta-MSH, neurotensin, NPY PHI, somatostatin and substance P on proliferation of lymphocytes *in vitro*. *Acta Physiol Scand* 1989; **137**: 107–111.
85. Karalis K, Mastorakos G, Chrousos GP, Tolis G. Somatostatin analogues suppress the inflammatory reaction *in vivo*. *J Clin Invest* 1994; **93**: 2000–2006.
86. Matucci-Cerinic M, Borrelli F, Generini S, *et al.* Somatostatin-induced modulation of inflammation in experimental arthritis. *Arthritis Rheum* 1995; **38**: 1687–1693.
87. Mulvihill SJ, Pappas TN, Fonkalsrud EW, Debas HT. The effect of somatostatin on experimental intestinal obstruction. *Ann Surg* 1988; **207**: 169–173.
88. Dorta G, Schnegg JF, Saraga E, Schmied PA. Treatment of gold-induced enteritis with octreotide. *Lancet* 1993; **342**: 179.
89. Benes G. Octreotide acetete (Sandostatin) to control the symptoms of Crohn's disease. *Am J Gastroenterol* 1993; **88**: 1603.
90. Kataeva G, Agro A, Stanisz AM. Substance-P-mediated intestinal inflammation: inhibitory effects of CP 96,345 and SMS 201-995. *Neuroimmunomodulation* 1994; **1**: 350–356.
91. Payer J, Huorka M, Duris I, *et al.* Plasma somatostatin levels in ulcerative colitis. *Hepato-Gastroenterol* 1994; **41**: 552–553.
92. Payer J, Huorka M, Duris I, Mikulecky M, Kratochvíl'ová H, Ondrejka P. Circadian rhythmicity and cross correlation of plasma gastrin, cortisol and somatostatin levels in ulcerative colitis patients and healthy subjects. *Hepato-Gastroenterol* 1993; **40**: 272–275.
93. Binimelis J, Webb SM, Monés J, *et al.* Circulating immunoreactive somatostatin in gastrointestinal diseases. Decrease after vagotomy and enhancement in active ulcerative colitis, irritable bowel syndrome, and duodenal ulcer. *Scand J Gastroenterol* 1987; **22**: 931–937.
94. Kyosola K, Penttila O, Salaspuro M. Rectal mucosal adrenergic innervation and enterochromaffin cells in ulcerative colitis. *Scand J Gastroenterol* 1977; **12**: 363–367.
95. Ahonen A, Kyosola K, Penttila O. Enterochromaffin cells and macrophages in ulcerative colitis and irritable colon. *Ann Clin Res* 1976; **8**: 1–7.
96. Calam J, Ghatei MA, Domin J, *et al.* Regional differences in concentrations of regulatory peptides in human colon mucosal biopsy. *Dig Dis Sci* 1989; **34**: 1193–1198.
97. Watanabe T, Kubota Y, Sawada T, Muto T. Distribution and quantification of somatostatin in inflammatory disease. *Dis Colon Rectum* 1992; **35**: 488–494.
98. Koch TR, Carney JA, Morris VA, Go VLW. Somatostatin in the idiopathic inflammatory bowel diseases. *Dis Colon Rectum* 1988; **31**: 198–203.
99. Yamamoto H, Morise K, Kusugami K, *et al.* Abnormal neuropeptide concentration in rectal mucosa of patients with inflammatory bowel disease. *J Gastroenterol* 1996; **31**: 525–532.

100. Eliakim R, Karmeli F, Rachmilewitz D. Decreased somatostatin (SS) generation by colonic mucosa in active ulcerative colitis. *Gastroenterology* 1991; **100**: A578.
101. Dawson J, Bryant MG, Bloom SR, Peters TJ. Gastrointestinal regulatory peptide storage granule abnormalities in jejunal mucosal diseases. *Gut* 1984; **25**: 636–643.
102. van Hagen PM, Krenning EP, Reubi JC, *et al.* Somatostatin analogue scintigraphy in granulomatous diseases. *Eur J Nucl Med* 1994; **21**: 497–502.
103. Reubi JC, Krenning EP, Lamberts SW, Laissue J. *In vitro* and *in vivo* detection of somatostatin receptors in human sarcoidosis. *Schweiz Med Wochenschr* 1992; **12**: 1636.
104. Lamberts SWJ, Krenning EP, Reubi JC. The role of somatostatin and its analogues in the diagnosis and treatment of tumors. *Endocrine Rev* 1991; **12**: 450–482.
105. Reubi J. Somatostatin receptors in the gastrointestinal tract in health and disease. *Yale J Biol Med* 1992; **65**: 493–503.
106. Reubi J, Horisberger U, Waser B, Gebbers J, Laissue J. High affinity somatostatin receptors in the gut-associated lymphoid tissues; preferential location in germinal centers. *Gastroenterology* 1992; **103**: 1207–1214.
107. Fehér E, Fodor M, Burnstock G. Distribution of somatostatin-immunoreactive nerve fibres in Peyer's patches. *Gut* 1992; **33**: 1195–1198.
108. Lundergan CF, Foegh ML, Ramwell PW. Peptide inhibition of myointimal proliferation by angiopetin, a somatostatin analogue. *J Am Coll Cardiol* 1991; **17**: 132B–136B.
109. Reubi JC, Mazzucchelli L, Laissue JA. Intestinal vessels express a high density of somatostatin receptors in human inflammatory bowel disease. *Gastroenterology* 1994; **106**: 951–959.
110. Reubi JC, Waser B, Markusse HM, Krenning EP, van Hagen M, Laissue JP. Vascular somatostatin receptors in synovium from patients with rheumatoid arthritis. *Eur J Pharmacol* 1994; **271**: 371–378.
111. Reubi JC, Horisberger U, Laissue JA. High density of somatostatin receptors in veins surrounding human cancer tissue: role in tumor-host interaction? *Int J Cancer* 1994; **56**: 681–688.
112. Reubi JC, Laissue J, Waser B, Horisberger U, Schaer J-C. Expression of somatostatin receptors in normal, inflamed, and neoplastic human gastrointestinal tissue. *Ann NY Acad Sci* 1994; **733**: 122–137.
113. Eliakim R, Karmeli F, Okon E, Rachmilewitz D. Octreotide effectively decreases mucosal damage in experimental colitis. *Gut* 1993; **34**: 264–269.
114. Agro A, Padaol I, Stanisz AM. Immunomodulatory activities of the somatostatin analogue BIM 23014c: effects on murine lymphocyte proliferation and natural killer activity. *Regul Pept* 1991; **32**: 129–139.
115. Elliott DE, Weinstock JV. Granulomas in murine *Schistosomiasis mansoni* have a somatostatin immunoregulatory circuit. *Metabolism* 1996; **45** (suppl 1): 88–90.
116. Teitelbaum DH, Del Valle J, Reyas B, *et al.* Intestinal intraepithelial lymphocytes influence the production of somatostatin. *Surgery* 1996; **120**: 227–233.

**Received 5 September 1997; accepted 5 September 1997**
